# The 20^th^ century: the dawn of modern neurotrauma treatment

**DOI:** 10.25122/jml-2024-1008

**Published:** 2024-05

**Authors:** Stefana-Andrada Dobran, Dafin Fior Muresanu

**Affiliations:** 1 RoNeuro Institute for Neurological Research and Diagnostic, Cluj-Napoca, Romania; 2 Department of Neuroscience, Iuliu Hatieganu University of Medicine and Pharmacy, Cluj-Napoca, Romania

The 20^th^ century was a transformative era for neurosurgery, a time when global conflicts and rapid societal changes necessitated significant medical breakthroughs. As the burden of neurotrauma was growing, practical solutions were needed. This period not only reshaped neurotrauma care through incredible innovation in medical practice, but also showcased the resourcefulness of humankind in the face of adversity.

## HARVEY CUSHING – THE FATHER OF MODERN NEUROSURGERY

**Figure 1:**
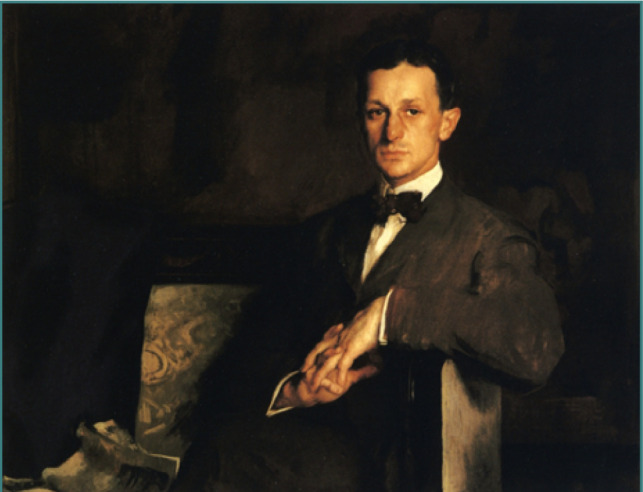
"Dr. Harvey Cushing," oil on canvas, by the American artist Edmund Tarbell. Available from: https://commons.wikimedia.org/wiki/File:Dr_Harvey_Cushing_Edmund_Tarbell_1908.jpeg

At the heart of this transformative age was Harvey William Cushing (1869-1939) (Figure 1), whose work established the very foundations of neurosurgery. In his seminal 1904 publication, ‘The Special Field of Neurological Surgery,’ Cushing established neurosurgery as a distinct surgical discipline and introduced surgery techniques that significantly decreased surgical risks associated with brain operations [1]. His ‘ether charts’ represented the first implementation of continuous monitoring. These charts documented the pulse, temperature, respiration, and, later on, blood pressure – an innovation that led to improved patient outcomes. Moreover, during the First World War, he used an electromagnet attached to a wire nail to remove metallic bullet fragments from the brain affected by gunshot wounds, showing remarkable creativity [2]. This, alongside his development of electrosurgical techniques and methods to reduce complications such as infections and the effects of intracranial pressure, greatly enhanced the survivability of neurosurgical patients [3].

His contributions to anesthesia were equally significant, as he was the first to introduce an independent neurosurgical anesthetist and developed new methods in regional anesthesia. Harvey Cushing significantly advanced neurosurgery by introducing intraoperative monitoring, which included blood pressure measurements and precordial auscultation [4]. His dedication to improving surgical outcomes was further demonstrated through his innovative approaches to pituitary surgery and his management of hemorrhage using adrenaline infiltrations. He also invented the ‘Cushing clip’ for better hemostasis and enhanced aseptic techniques in the operating room [2,5].

Educated at Yale and Harvard, Cushing's rigorous academic background fueled his prolific research career, leading to his recognition as a Pulitzer Prize winner for his biography ‘Life of Sir William Osler’ and as a member of numerous prestigious medical societies. He left a significant legacy, founding a school of neurosurgery, introducing the documentation of the clinical and pathological details of cerebral tumors, and developing operative surgery techniques, among many other advancements [2].

The father of modern neurosurgery shaped the practice through his dedication and innovative mind, even in the face of criticism and adversity, stressing the importance of patient care and setting the groundwork for further developments that will forever transform neurosurgery from a rudimentary practice to a specialty that intertwines science, innovation, and dedication to improving patients’ lives.

## GLASGOW COMA SCALE: THE GOLD STANDARD FOR EVALUATING CONSCIOUSNESS

The Glasgow Coma Scale (GCS), developed in 1974 by University of Glasgow Professors Sir Graham Teasdale and Bryan Jennet, dramatically transformed the evaluation of traumatic brain injury (TBI) [6]. This scale quickly became a cornerstone in emergency rooms and intensive care units globally, providing a systematic method to assess the severity of neurotrauma, thus enhancing patient care by reducing mortality, preventing further injury, and improving quality of life. Originally designed to evaluate consciousness levels in patients with head injuries, the GCS has become integral to neurotrauma management, offering clear, objective criteria that previously lacked consistency. Its simplicity and practicality for assessing both traumatic and non-traumatic comas have made it the international standard for determining patient prognosis and monitoring ongoing conditions in acute care settings [1,6].

The publication introducing the GCS, “Assessment of Coma and Impaired Consciousness, A Practical Scale,” has gathered over 10,000 citations, reflecting its widespread influence and critical role in neurology [7]. The prevalent adoption of this tool has reshaped neurotrauma assessment during an era defined by advancements in medical response to war injuries and a shortage of neurosurgeons, providing reliable data that has improved survival rates and patient outcomes [8].

## MEDICINE AT WAR: NEUROTRAUMA ON THE BATTLEFIELDS

**Figure 2:**
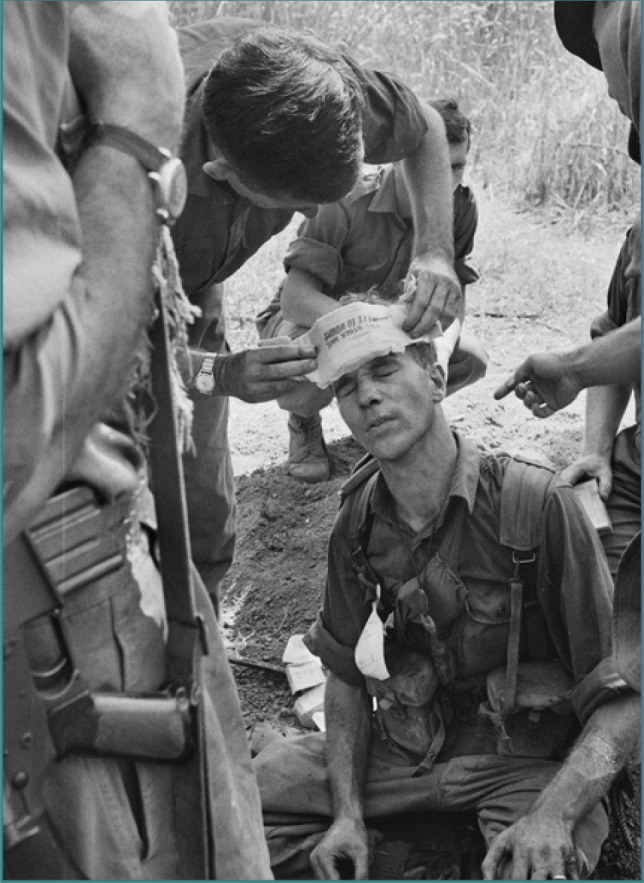
1RAR soldier is treated for head wound during Operation Silver City, March 1966. Available from: https://commons.wikimedia.org/wiki/File:1RAR_soldier_is_treated_for_head_wound_during_Operation_Silver_City,_March_1966.jpg

Historically, the 20^th^ century has been marked by significant political conflicts, such as the two World Wars, the Vietnam War, the Korean War, and the US Civil War (Figure 2). As a silver lining to those challenging times, remarkable progress has been achieved in neurotrauma management.

During the First World War, the necessity to manage brain injuries led to significant developments in surgical techniques and the use of antibiotics, which substantially reduced mortality rates. This period also saw neurologists like Gordon Holmes and neurosurgeons such as Percy Sargent make pivotal contributions to understanding and treating intracranial pressure, which significantly advanced the field of brain injury management [9].

In 1996, US President Bill Clinton signed the Traumatic Brain Injury Act into law - the only federal legislation specifically addressing prevention, research, and service delivery through grants to states. The key federal agencies involved were the Centers for Disease Control and Prevention (CDC), the National Institutes of Health (NIH), and the Administration for Community Living (ACL) [10].

A consequence of the widespread political conflicts represented the development of tactical neurocritical care for patients with central nervous system injuries from the battlefield and the initiation of the first intensive care unit by Dr. Walter E. Dandy. In 1923, Dr. Dandy started a three-bed postoperative neurosurgical intensive care unit at Johns Hopkins Hospital in the USA, where nurses cared for patients under the supervision of surgeons. These units proved essential during the Second World War, a period in which the hospitals witnessed the introduction of ICU units [9]. Other reputable institutions later followed his lead.

The wars led to innovations in neurotrauma management, laying the ground for neurosurgery as a medical specialty. Among the progress, we mention the early operation of trauma, mobile neurosurgical units, improved resuscitation, cranioplasty, external drainage systems following cranial trauma, infection prevention, and multidisciplinary care, alongside new strategies, guidelines, and improved standards of practice [11].

## NEUROTRAUMA IN THE 20^TH^ CENTURY: TO BE CONTINUED

The 20^th^ century marked an era of unprecedented advances in neurotrauma, characterized by groundbreaking innovations and shifting paradigms. As we look to the future, our editorial series will continue to explore the dynamic field of neurotrauma treatment. Our next editorial will explore the evolution of spinal trauma and imaging, with special consideration to some of the most important innovations in the field so far - computed tomography (CT), magnetic resonance imaging (MRI), intracranial pressure (ICP) monitoring, and fluoroscopy — techniques that forever changed the evaluation of neurotrauma as well as practices in neurosurgery. Moreover, we will delve into the fascinating field of robotics which will demonstrate how technological advancements shape medicine.

Our journey will explore how sports shaped medicine and vice-versa and help us get a better understanding of the concept of chronic traumatic encephalopathy. Further on, we will explore how societal progress, following the Industrial Revolution, shaped the story of neurotrauma. Lastly, we will pay homage to the last decade of the 20^th^ century - what is known as the ‘Decade of the Brain’.

The lessons of the 20^th^ century have taught us that adversity is a catalyst for innovation and that the simplest solutions are often the most profound. The history of neurotrauma shows us again and again that, when faced with obstacles, human creativity and resilience can overcome the most dire challenges. As we reflect on these developments, it becomes evident that neurotraumatology, while sometimes underappreciated within the broader domain of neuroscience, offers a fascinating narrative of progress and possibility.

## References

[1] Bertullo G (2015). History of Traumatic Brain Injury (TBI). American Journal of BioMedicine.

[2] Ellis H (2009). Harvey Cushing: a founding father of neurosurgery. Br J Hosp Med (Lond).

[3] Voorhees JR, Cohen-Gadol AA, Laws ER, Spencer DD (2005). Battling blood loss in neurosurgery: Harvey Cushing's embrace of electrosurgery. J Neurosurg.

[4] Molnár C, Nemes C, Szabó S, Fülesdi B (2008). Harvey Cushing, a pioneer of neuroanesthesia. J Anesth.

[5] ElSaban M, Bhatt G, Lee J, Koshiya H, Mansoor T, Amal T, Kashyap R (2023). A historical delve into neurotrauma-focused critical care. Wiener Medizinische Wochenschrift.

[6] Jain S (2023). Glasgow Coma Scale. StatPearls [Internet].

[7] Aguilar-Fuentes V, Orozco-Puga P, Jiménez-Ruiz A (2024). The Glasgow Coma Scale: 50-year anniversary. Neurol Sci.

[8] Mattei TA, Teasdale GM (2020). The Story of the Development and Adoption of the Glasgow Coma Scale: Part I, The Early Years. World Neurosurg.

[9] ElSaban M, Bhatt G, Lee J, Koshiya H, Mansoor T, Amal T, Kashyap R (2023). A historical delve into neurotrauma-focused critical care. Wien Med Wochenschr.

[10] Traumatic brain injury (TBI) act (2022). Brain Injury Association of America. https://www.biausa.org/public-affairs/public-policy/traumatic-brain-injury-act.

[11] Rainone GJ, Zelmanovich R, Laurent D, Lucke-Wold B (2023). How War Has Shaped Neurosurgery. World Neurosurg.

